# (*E*)-1-{6-[1-(2,6-Dimethyl­phenyl­imino)­eth­yl]pyridin-2-yl}ethanone

**DOI:** 10.1107/S1600536811056327

**Published:** 2012-01-11

**Authors:** Qing Su, Qing Zhao

**Affiliations:** a School of Chemistry, Jilin University, Changchun 130012, People’s Republic of China; b Department of Neurology, China-Japan Union Hospital, Jilin University, Changchun 130033, People’s Republic of China

## Abstract

In the title compound, C_17_H_18_N_2_O, the dijedral angle between the mean planes of the pyridine and benzene rings is 78.0 (1)°. In the crystal, pairs of C—H⋯O inter­actions with graph-set motif *R*_2_^2^(10) form inversion dimers. Adjacent dimers are further connected into a three-dimensional network by C—H⋯O connections. There is also an inter­action between the carbonyl groups in adjacent mol­ecules with an O⋯C distance of 3.176 (2) Å.

## Related literature

For the synthesis of mono- and bis­(imino)­pyridine ligands and catalytic applications of their metal complexes, see: Schmidt *et al.* (2002 ▶); Bianchini *et al.* (2003 ▶); Britovsek *et al.* (1999 ▶); Mecking *et al.* (2001 ▶); Gibson *et al.* (2007 ▶). For graph-set analysis of hydrogen-bonded networks, see: Bernstein *et al.* (1995 ▶). For carbon­yl–carbonyl inter­actions, see: Allen *et al.* (1998 ▶).
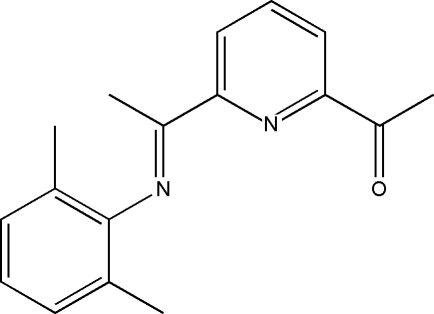


## Experimental

### 

#### Crystal data

C_17_H_18_N_2_O
*M* *_r_* = 266.33
Triclinic, 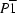

*a* = 6.2988 (13) Å
*b* = 7.9684 (16) Å
*c* = 16.009 (3) Å
α = 99.57 (3)°
β = 96.40 (3)°
γ = 108.31 (3)°
*V* = 740.6 (3) Å^3^
*Z* = 2
Mo *K*α radiation
μ = 0.08 mm^−1^
*T* = 293 K
0.48 × 0.39 × 0.21 mm

#### Data collection

Rigaku R-AXIS RAPID diffractometer
Absorption correction: multi-scan (*ABSCOR*; Higashi, 1995 ▶) *T*_min_ = 0.965, *T*_max_ = 0.984
7308 measured reflections
3359 independent reflections
2372 reflections with *I* > 2σ(*I*)
*R*_int_ = 0.017

#### Refinement

*R*[*F* ^2^ > 2σ(*F* ^2^)] = 0.048
*wR*(*F* ^2^) = 0.143
*S* = 1.06
3359 reflections
185 parameters
H-atom parameters constrained
Δρ_max_ = 0.26 e Å^−3^
Δρ_min_ = −0.13 e Å^−3^

### 

Data collection: *RAPID-AUTO* (Rigaku, 1998 ▶); cell refinement: *RAPID-AUTO*; data reduction: *RAPID-AUTO*; program(s) used to solve structure: *SHELXS97* (Sheldrick, 2008 ▶); program(s) used to refine structure: *SHELXL97* (Sheldrick, 2008 ▶); molecular graphics: *SHELXTL* (Sheldrick, 2008 ▶); software used to prepare material for publication: *SHELXTL*.

## Supplementary Material

Crystal structure: contains datablock(s) global, I. DOI: 10.1107/S1600536811056327/mw2044sup1.cif

Structure factors: contains datablock(s) I. DOI: 10.1107/S1600536811056327/mw2044Isup2.hkl

Supplementary material file. DOI: 10.1107/S1600536811056327/mw2044Isup3.cml

Additional supplementary materials:  crystallographic information; 3D view; checkCIF report

## Figures and Tables

**Table 1:** Hydrogen-bond geometry (Å, °)

*D*—H⋯*A*	*D*—H	H⋯*A*	*D*⋯*A*	*D*—H⋯*A*
C2—H2⋯O1^i^	0.93	2.64	3.459 (2)	147
C9—H9*C*⋯O1^ii^	0.96	2.59	3.366 (2)	138

Symmetry codes: (i) 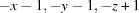; (ii) 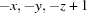.

**Fig. 1.**
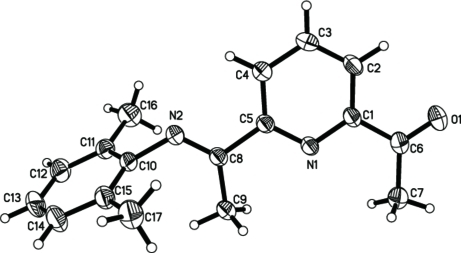
View of the molecule of (I) showing the atom labelling scheme. Displacement ellipsoids are drawn at the 30% probability level.

**Fig. 2.**
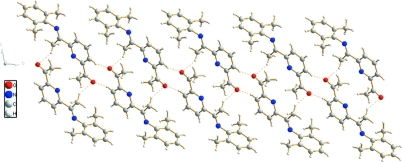
The molecular packing of (I). Hydrogen bonds are indicated by dashed lines.

## supplementary crystallographic information

### Comment

Bis(imino)pyridine iron and cobalt complexes have been well-known as catalyst precursors for olefin oligomerization and polymerization. Considerable efforts have been focused on improving catalyst performance with a view to enhancing catalytic activity and control of the microstructure of the resulting polymer. (Britovsek, *et al.*, 1999; Mecking, *et al.*, 2001; Gibson, *et al.*, 2007;) The nature of the bis(imino)pyridine ligands was found to be a crucial factor affecting the catalyst performance and it has also been shown that similar complexes of mono(imino)pyridine ligands can function as active catalysts (Bianchini *et al.*, 2003).

In the title molecule, Fig. 1, the angle between the mean planes of the pyridine and benzene rings is 78.04 (6)°. In the crystal, there exist intermolecular C2—H2···O1 interactions with the graph-set motif *R*_2_^2^(10) (Bernstein *et al.*, 1995) which form a dimer (Fig.2 and Table 1). The adjacent dimers are connected into a 3-dimensional network by intermolecular C9—H9C···O1 interactions (Fig.2 and Table 1), and significant interactions between centrosymmetrically-related pairs of carbonyl groups (Allen, *et al.*, 1998) in adjacent molecules with an O1···C6^iii^ distance of 3.176 (2) Å and a C6=O1···C6^iii^ angle of 93.48 (9)° [(iii) -x, -1-y, 1-z].

### Experimental

Compound (I) was prepared as described in the litererature (Schmidt *et al.*, 2002; Bianchini *et al.*, 2003) with 2,6-diacetylpyridine and 2,6-dimethylaniline as starting material. Crystals suitable for X-ray analysis were obtained by recrystallization from a petroleum ether solution at room temperature.

### Refinement

The C-bound H atoms were positioned geometrically with C—H = 0.93 Å (aromatic carbon), and 0.96 (methyl) Å, and allowed to ride on their parent atoms in the riding model approximation with *U*_iso_(H) = 1.2 (1.5 for methyl) *U*_eq_(C).

### Crystal data

**Table:**

C_17_H_18_N_2_O	*Z* = 2
*M**_r_* = 266.33	*F*(000) = 284
Triclinic, *P*1	*D*_x_ = 1.194 Mg m^−^^3^
Hall symbol: -P 1	Mo *K*α radiation, λ = 0.71073 Å
*a* = 6.2988 (13) Å	Cell parameters from 5656 reflections
*b* = 7.9684 (16) Å	θ = 3.3–27.5°
*c* = 16.009 (3) Å	µ = 0.08 mm^−^^1^
α = 99.57 (3)°	*T* = 293 K
β = 96.40 (3)°	Block, yellow
γ = 108.31 (3)°	0.48 × 0.39 × 0.21 mm
*V* = 740.6 (3) Å^3^	

### Data collection

**Table:**

Rigaku R-AXIS RAPID diffractometer	3359 independent reflections
Radiation source: fine-focus sealed tube	2372 reflections with *I* > 2σ(*I*)
graphite	*R*_int_ = 0.017
ω scans	θ_max_ = 27.5°, θ_min_ = 3.3°
Absorption correction: multi-scan (*ABSCOR*; Higashi, 1995)	*h* = −8→8
*T*_min_ = 0.965, *T*_max_ = 0.984	*k* = −10→10
7308 measured reflections	*l* = −20→20

### Refinement

**Table:**

Refinement on *F*^2^	Primary atom site location: structure-invariant direct methods
Least-squares matrix: full	Secondary atom site location: difference Fourier map
*R*[*F*^2^ > 2σ(*F*^2^)] = 0.048	Hydrogen site location: inferred from neighbouring sites
*wR*(*F*^2^) = 0.143	H-atom parameters constrained
*S* = 1.06	*w* = 1/[σ^2^(*F*_o_^2^) + (0.080*P*)^2^ + 0.0412*P*] where *P* = (*F*_o_^2^ + 2*F*_c_^2^)/3
3359 reflections	(Δ/σ)_max_ < 0.001
185 parameters	Δρ_max_ = 0.26 e Å^−^^3^
0 restraints	Δρ_min_ = −0.13 e Å^−^^3^

### Special details

**Table:**

Geometry. All e.s.d.'s (except the e.s.d. in the dihedral angle between two l.s. planes) are estimated using the full covariance matrix. The cell e.s.d.'s are taken into account individually in the estimation of e.s.d.'s in distances, angles and torsion angles; correlations between e.s.d.'s in cell parameters are only used when they are defined by crystal symmetry. An approximate (isotropic) treatment of cell e.s.d.'s is used for estimating e.s.d.'s involving l.s. planes.
Refinement. Refinement of *F*^2^ against ALL reflections. The weighted *R*-factor *wR* and goodness of fit *S* are based on *F*^2^, conventional *R*-factors *R* are based on *F*, with *F* set to zero for negative *F*^2^. The threshold expression of *F*^2^ > σ(*F*^2^) is used only for calculating *R*-factors(gt) *etc*. and is not relevant to the choice of reflections for refinement. *R*-factors based on *F*^2^ are statistically about twice as large as those based on *F*, and *R*- factors based on ALL data will be even larger.

### Fractional atomic coordinates and isotropic or equivalent isotropic displacement parameters (Å^2^)

**Table:**

	*x*	*y*	*z*	*U*_iso_*/*U*_eq_	
O1	−0.1379 (2)	−0.37717 (16)	0.54963 (7)	0.0681 (3)	
N1	−0.01368 (18)	−0.15963 (14)	0.37828 (7)	0.0422 (3)	
N2	−0.0626 (2)	0.02226 (15)	0.19274 (7)	0.0471 (3)	
C1	−0.1418 (2)	−0.27314 (17)	0.42051 (9)	0.0434 (3)	
C2	−0.3694 (2)	−0.37081 (19)	0.39062 (10)	0.0531 (4)	
H2	−0.4536	−0.4474	0.4220	0.064*	
C3	−0.4679 (3)	−0.3518 (2)	0.31350 (11)	0.0607 (4)	
H3	−0.6201	−0.4171	0.2914	0.073*	
C4	−0.3396 (2)	−0.23533 (19)	0.26906 (10)	0.0524 (4)	
H4	−0.4038	−0.2206	0.2168	0.063*	
C5	−0.1126 (2)	−0.14023 (16)	0.30375 (8)	0.0410 (3)	
C6	−0.0265 (3)	−0.28860 (19)	0.50480 (9)	0.0494 (3)	
C7	0.2229 (3)	−0.1950 (2)	0.53064 (10)	0.0616 (4)	
H7A	0.2725	−0.2148	0.5859	0.092*	
H7B	0.2578	−0.0676	0.5338	0.092*	
H7C	0.2994	−0.2421	0.4889	0.092*	
C8	0.0334 (2)	−0.00400 (16)	0.26111 (8)	0.0410 (3)	
C9	0.2751 (2)	0.0919 (2)	0.30375 (10)	0.0543 (4)	
H9A	0.3439	0.1871	0.2753	0.081*	
H9B	0.3561	0.0078	0.3003	0.081*	
H9C	0.2806	0.1426	0.3630	0.081*	
C10	0.0553 (2)	0.15611 (17)	0.14995 (8)	0.0445 (3)	
C11	0.0037 (2)	0.31576 (18)	0.15929 (9)	0.0496 (3)	
C12	0.1034 (3)	0.4428 (2)	0.11335 (11)	0.0632 (4)	
H12	0.0720	0.5502	0.1193	0.076*	
C13	0.2480 (3)	0.4132 (2)	0.05903 (12)	0.0746 (5)	
H13	0.3128	0.4997	0.0283	0.090*	
C14	0.2970 (3)	0.2554 (3)	0.05017 (12)	0.0724 (5)	
H14	0.3953	0.2366	0.0133	0.087*	
C15	0.2030 (3)	0.1238 (2)	0.09492 (9)	0.0545 (4)	
C16	−0.1539 (3)	0.3483 (2)	0.21889 (13)	0.0713 (5)	
H16A	−0.0827	0.3648	0.2773	0.107*	
H16B	−0.2920	0.2460	0.2060	0.107*	
H16C	−0.1879	0.4547	0.2114	0.107*	
C17	0.2582 (4)	−0.0483 (2)	0.08336 (12)	0.0711 (5)	
H17A	0.2760	−0.0826	0.0249	0.107*	
H17B	0.1370	−0.1427	0.0966	0.107*	
H17C	0.3968	−0.0294	0.1212	0.107*	

### Atomic displacement parameters (Å^2^)

**Table:**

	*U*^11^	*U*^22^	*U*^33^	*U*^12^	*U*^13^	*U*^23^
O1	0.0754 (8)	0.0750 (7)	0.0612 (6)	0.0197 (6)	0.0261 (6)	0.0361 (6)
N1	0.0402 (6)	0.0407 (6)	0.0481 (6)	0.0116 (5)	0.0132 (5)	0.0164 (5)
N2	0.0446 (6)	0.0447 (6)	0.0520 (6)	0.0100 (5)	0.0094 (5)	0.0201 (5)
C1	0.0459 (7)	0.0389 (6)	0.0500 (7)	0.0144 (6)	0.0172 (6)	0.0162 (5)
C2	0.0467 (8)	0.0496 (8)	0.0653 (9)	0.0088 (6)	0.0208 (7)	0.0255 (7)
C3	0.0391 (8)	0.0600 (9)	0.0748 (10)	0.0014 (6)	0.0079 (7)	0.0239 (8)
C4	0.0440 (8)	0.0520 (8)	0.0586 (8)	0.0087 (6)	0.0061 (6)	0.0217 (7)
C5	0.0396 (7)	0.0381 (6)	0.0473 (7)	0.0121 (5)	0.0119 (5)	0.0140 (5)
C6	0.0586 (9)	0.0457 (7)	0.0500 (7)	0.0195 (6)	0.0178 (6)	0.0174 (6)
C7	0.0593 (10)	0.0645 (9)	0.0594 (9)	0.0157 (8)	0.0052 (7)	0.0225 (7)
C8	0.0399 (7)	0.0380 (6)	0.0467 (7)	0.0116 (5)	0.0121 (5)	0.0133 (5)
C9	0.0433 (8)	0.0586 (8)	0.0565 (8)	0.0048 (6)	0.0096 (6)	0.0236 (7)
C10	0.0421 (7)	0.0430 (7)	0.0462 (7)	0.0081 (6)	0.0054 (6)	0.0177 (6)
C11	0.0490 (8)	0.0449 (7)	0.0533 (8)	0.0125 (6)	0.0049 (6)	0.0155 (6)
C12	0.0712 (11)	0.0462 (8)	0.0739 (10)	0.0168 (7)	0.0091 (8)	0.0255 (7)
C13	0.0807 (13)	0.0666 (11)	0.0852 (12)	0.0158 (9)	0.0293 (10)	0.0481 (9)
C14	0.0790 (12)	0.0786 (11)	0.0771 (11)	0.0297 (10)	0.0398 (10)	0.0411 (9)
C15	0.0587 (9)	0.0548 (8)	0.0557 (8)	0.0198 (7)	0.0171 (7)	0.0221 (7)
C16	0.0746 (12)	0.0665 (10)	0.0844 (12)	0.0338 (9)	0.0261 (9)	0.0204 (9)
C17	0.0866 (13)	0.0702 (11)	0.0733 (11)	0.0400 (10)	0.0308 (9)	0.0236 (9)

### Geometric parameters (Å, °)

**Table:**

O1—C6	1.2147 (17)	C9—H9B	0.9600
N1—C5	1.3386 (18)	C9—H9C	0.9600
N1—C1	1.3388 (16)	C10—C11	1.3977 (19)
N2—C8	1.2712 (17)	C10—C15	1.4022 (19)
N2—C10	1.4202 (16)	C11—C12	1.382 (2)
C1—C2	1.383 (2)	C11—C16	1.501 (2)
C1—C6	1.502 (2)	C12—C13	1.373 (3)
C2—C3	1.372 (2)	C12—H12	0.9300
C2—H2	0.9300	C13—C14	1.375 (3)
C3—C4	1.377 (2)	C13—H13	0.9300
C3—H3	0.9300	C14—C15	1.384 (2)
C4—C5	1.389 (2)	C14—H14	0.9300
C4—H4	0.9300	C15—C17	1.506 (2)
C5—C8	1.4997 (17)	C16—H16A	0.9600
C6—C7	1.487 (2)	C16—H16B	0.9600
C7—H7A	0.9600	C16—H16C	0.9600
C7—H7B	0.9600	C17—H17A	0.9600
C7—H7C	0.9600	C17—H17B	0.9600
C8—C9	1.494 (2)	C17—H17C	0.9600
C9—H9A	0.9600		
			
C5—N1—C1	117.90 (12)	H9A—C9—H9C	109.5
C8—N2—C10	121.37 (12)	H9B—C9—H9C	109.5
N1—C1—C2	123.17 (13)	C11—C10—C15	121.17 (12)
N1—C1—C6	116.46 (12)	C11—C10—N2	117.17 (12)
C2—C1—C6	120.37 (12)	C15—C10—N2	121.46 (12)
C3—C2—C1	118.35 (13)	C12—C11—C10	118.41 (14)
C3—C2—H2	120.8	C12—C11—C16	121.22 (14)
C1—C2—H2	120.8	C10—C11—C16	120.37 (13)
C2—C3—C4	119.49 (14)	C13—C12—C11	121.19 (15)
C2—C3—H3	120.3	C13—C12—H12	119.4
C4—C3—H3	120.3	C11—C12—H12	119.4
C3—C4—C5	118.80 (14)	C12—C13—C14	119.88 (14)
C3—C4—H4	120.6	C12—C13—H13	120.1
C5—C4—H4	120.6	C14—C13—H13	120.1
N1—C5—C4	122.27 (12)	C13—C14—C15	121.44 (15)
N1—C5—C8	116.15 (12)	C13—C14—H14	119.3
C4—C5—C8	121.54 (12)	C15—C14—H14	119.3
O1—C6—C7	121.86 (14)	C14—C15—C10	117.91 (14)
O1—C6—C1	119.54 (14)	C14—C15—C17	120.26 (14)
C7—C6—C1	118.60 (12)	C10—C15—C17	121.82 (13)
C6—C7—H7A	109.5	C11—C16—H16A	109.5
C6—C7—H7B	109.5	C11—C16—H16B	109.5
H7A—C7—H7B	109.5	H16A—C16—H16B	109.5
C6—C7—H7C	109.5	C11—C16—H16C	109.5
H7A—C7—H7C	109.5	H16A—C16—H16C	109.5
H7B—C7—H7C	109.5	H16B—C16—H16C	109.5
N2—C8—C9	125.99 (12)	C15—C17—H17A	109.5
N2—C8—C5	116.51 (12)	C15—C17—H17B	109.5
C9—C8—C5	117.47 (12)	H17A—C17—H17B	109.5
C8—C9—H9A	109.5	C15—C17—H17C	109.5
C8—C9—H9B	109.5	H17A—C17—H17C	109.5
H9A—C9—H9B	109.5	H17B—C17—H17C	109.5
C8—C9—H9C	109.5		
			
C5—N1—C1—C2	0.26 (19)	N1—C5—C8—C9	1.94 (17)
C5—N1—C1—C6	−178.75 (11)	C4—C5—C8—C9	179.65 (12)
N1—C1—C2—C3	0.7 (2)	C8—N2—C10—C11	−104.44 (15)
C6—C1—C2—C3	179.71 (13)	C8—N2—C10—C15	80.74 (18)
C1—C2—C3—C4	−0.9 (2)	C15—C10—C11—C12	−0.5 (2)
C2—C3—C4—C5	0.2 (2)	N2—C10—C11—C12	−175.34 (13)
C1—N1—C5—C4	−1.06 (19)	C15—C10—C11—C16	−179.67 (15)
C1—N1—C5—C8	176.62 (10)	N2—C10—C11—C16	5.5 (2)
C3—C4—C5—N1	0.9 (2)	C10—C11—C12—C13	0.7 (2)
C3—C4—C5—C8	−176.71 (12)	C16—C11—C12—C13	179.82 (18)
N1—C1—C6—O1	173.71 (12)	C11—C12—C13—C14	−0.5 (3)
C2—C1—C6—O1	−5.3 (2)	C12—C13—C14—C15	0.1 (3)
N1—C1—C6—C7	−6.63 (18)	C13—C14—C15—C10	0.0 (3)
C2—C1—C6—C7	174.33 (13)	C13—C14—C15—C17	179.49 (18)
C10—N2—C8—C9	−2.1 (2)	C11—C10—C15—C14	0.2 (2)
C10—N2—C8—C5	176.01 (11)	N2—C10—C15—C14	174.78 (15)
N1—C5—C8—N2	−176.33 (11)	C11—C10—C15—C17	−179.28 (15)
C4—C5—C8—N2	1.38 (18)	N2—C10—C15—C17	−4.7 (2)

### Hydrogen-bond geometry (Å, °)

**Table:**

*D*—H···*A*	*D*—H	H···*A*	*D*···*A*	*D*—H···*A*
C2—H2···O1^i^	0.93	2.64	3.459 (2)	147
C9—H9C···O1^ii^	0.96	2.59	3.366 (2)	138

Symmetry codes: (i) −*x*−1, −*y*−1, −*z*+1; (ii) −*x*, −*y*, −*z*+1.
